# Risk of malaria in young children after periconceptional iron supplementation

**DOI:** 10.1111/mcn.13106

**Published:** 2020-11-25

**Authors:** Sabine Gies, Stephen A. Roberts, Salou Diallo, Olga M. Lompo, Halidou Tinto, Bernard J. Brabin

**Affiliations:** ^1^ Department of Biomedical Sciences Prince Leopold Institute of Tropical Medicine Antwerp Belgium; ^2^ Medical Mission Institute Wurzburg Germany; ^3^ Centre for Biostatistics, Division of Population Health, Health Services Research and Primary Care, Faculty of Biology, Medicine and Health University of Manchester, Manchester Academic Health Science Centre (MAHSC) Manchester UK; ^4^ Institute for Research in Health Sciences–Clinical Research Unit of Nanoro (IRSS–URCN) Ouagadougou Burkina Faso; ^5^ Service d'Anatomocytopathologie et de Médicine Légale Centre Hospitalier Universitaire Yalgado Ouedraogo Ouagadougou Burkina Faso; ^6^ Clinical Division Liverpool School of Tropical Medicine Liverpool UK; ^7^ Institute of Infection and Global Health University of Liverpool Liverpool UK; ^8^ Global Child Health Group, Academic Medical Centre University of Amsterdam Amsterdam The Netherlands

**Keywords:** Burkina Faso, child, iron, malaria, periconceptional, placenta

## Abstract

This study in Burkina Faso investigated whether offspring of young mothers who had received weekly periconceptional iron supplementation in a randomised controlled trial were at increased risk of malaria. A child safety survey was undertaken in the peak month of malaria transmission towards the end of the trial to assess child iron biomarkers, nutritional status, anaemia and malaria outcomes. Antenatal iron biomarkers, preterm birth, fetal growth restriction and placental pathology for malaria and chorioamnionitis were assessed. Data were available for 180 babies surviving to the time of the survey when their median age was 9 months. Prevalence of maternal iron deficiency in the last trimester based on low body iron stores was 16%. Prevalence of active placental malaria infection was 24.8%, past infection 59% and chorioamnionitis 55.6%. Babies of iron supplemented women had lower median gestational age. Four out of five children ≥ 6 months were iron deficient, and 98% were anaemic. At 4 months malaria prevalence was 45%. Child iron biomarkers, anaemia and malaria outcomes did not differ by trial arm. Factors associated with childhood parasitaemia were third trimester C‐reactive protein level (OR 2.1; 95% CI 1.1–3.9), active placental malaria (OR 5.8; 1.0–32.5, *P* = 0.042) and child body iron stores (OR 1.13; 1.04–1.23, *P* = 0.002). Chorioamnionitis was associated with reduced risk of child parasitaemia (OR 0.4; 0.1–1.0, *P* = 0.038). Periconceptional iron supplementation of young women did not alter body iron stores of their children. Higher child body iron stores and placental malaria increased risk of childhood parasitaemia.

**Key messages**

- Iron deficiency prevalence in young mothers in Burkina Faso was low.
- Periconceptional iron supplements did not alter placental or child malaria risk.
- Malaria parasitaemia risk was higher in children with better iron status.
- Placental malaria increased risk of child malaria.
- Intensified efforts are needed to reduce risk of placental malaria which should, in turn, reduce risk of malaria and anaemia in young children.

## 1 INTRODUCTION

Better iron status has been associated with increased *Plasmodium falciparum* infection risk early in pregnancy (Diallo et al., 2020; Moya‐Alvarez et al., 2015) and iron deficiency at delivery with decreased prevalence of placental malaria (Kabyemela, Fried, Kurtis, Mutabingwa, & Duffy, 2008; Senga, Harper, Koshy, Kazembe, & Brabin, 2011), suggesting a direct effect of maternal iron status on placental iron homeostasis. Placental adaptation, allowing optimal transfer of iron from the maternal circulation to the fetus (Sangkhae et al., 2020; Scholl, 2011), may be impaired by malaria. Malaria in pregnancy is also reported as a predictor of infant haemoglobin (Hb) levels (Accrombessi et al., 2015; Le Cessie et al., 2002).

Cohort studies from Benin (Moya‐Alvarez et al., 2017), Malawi (Jonker et al., 2012), Tanzania (Gwamaka et al., 2012), Zambia (Barffour et al., 2017), Kenya and Uganda (Muriuki et al., 2019) have reported that better iron status in young children predicted increased future malaria risk, with iron deficiency significantly decreasing odds of subsequent parasitaemia. A twofold increased risk in young children of malaria parasitaemia and clinical malaria was also seen in mothers experiencing *P. falciparum* infections during pregnancy (Park et al., 2020), with several studies identifying an association of increased child malaria with placental malaria in their mothers (Agbota, Accrombessi, et al., 2019; Asante et al., 2013; Awine et al., 2016; Bardají et al., 2011; Le Port et al., 2011; Schwarz et al., 2008; Sylvester et al., 2018; Sylvester et al., 2016). Given the protective effect of iron deficiency during pregnancy for malaria (Diallo et al., 2020; Kabyemela, Fried, Kurtis, Mutabingwa, & Duffy, 2008; Moya‐Alvarez et al., 2015; Senga, Harper, Koshy, Kazembe, & Brabin, 2011), it follows that young children born to iron replete mothers, enhanced by routine periconceptional or antenatal iron supplementation, could be at higher risk of malaria, although the issue is complex as immunological mechanisms (Brickley et al., 2015; Broen, Brustoski, Engelmann, & Luty, 2007; Dechavanne et al., 2017; Feeney, 2020; Hviid, 2009; Hviid & Staalsoe, 2004; Park et al., 2019; Sylvester et al., 2018) and maternal factors such as breast feeding and use of antimalarial drugs are relevant (Hawking, 1954; Kakuru, Staedke, Dorsey, Rogerson, & Chandramohan, 2019; Natama, Rovira‐Vallbona, Sorgho, et al., 2018).

To determine childhood malaria risk in relation to maternal and offspring iron status a cohort of children born to mothers who participated in a randomised double blind controlled safety trial of periconceptional weekly iron supplementation (Gies et al., 2018) was assessed during the peak month of malaria transmission towards the end of the trial. A child safety survey became necessary after publication in 2012 (post trial commencement) of two cohort studies that reported iron repletion predicted increased malaria risk in pre‐school children in Malawi (Jonker et al., 2012) and Tanzania (Gwamaka et al., 2012). The objectives of this cross‐sectional child safety study were firstly, in children under 2 years of age, to determine prespecified malaria‐related outcomes and anaemia and iron biomarkers by trial arm, and secondly, to characterise maternal and child iron status and related factors associated with child malaria.

## 2 MATERIALS AND METHODS

Written, informed consents were given by all individuals, with additional guardian consents provided for minors. The work described was carried out in accordance with the Code of Ethics of the World Medical Association (Declaration of Helsinki). The primary trial outcome was malaria prevalence at the first antenatal visit, but women were followed till delivery and gestational age, preterm birth, birthweight, placental malaria and neonatal deaths have been reported (Gies et al., 2018). The survey in children was introduced as a safety amendment to the registered trial protocol with a prespecified secondary outcome of malaria parasite prevalence. The main results of the study were communicated to communities at the end of the study.

### 2.1 Trial procedures

The main trial was undertaken between April 2011 and January 2014 in the rural area of Nanoro in Burkina Faso where malaria is hyperendemic with seasonal transmission. The study participants were enrolled within the Nanoro Health and Demographic Surveillance catchment area which had a population of approximately 55000 inhabitants. HIV prevalence in this population was 1.2% in women aged 15–49 years and 0.76% among pregnant women (Institut National de la Statistique et de la Démographie [INSD] et ICF International, 2012). Background and published data on the trial, randomisation and design (Brabin, Gies, et al., 2019; Diallo et al., 2020; Gies et al., 2018) are summarised in Appendix S1.

### 2.2 Pregnancy assessments

Nulliparous participants had been individually randomised to receive, directly observed, either a weekly capsule containing ferrous gluconate (60 mg) and folic acid (2.8 mg) (*n* = 980), or an identical capsule containing folic acid alone (2.8 mg) (*n* = 979) for up to 18 months, or till pregnancy occurred (Gies et al., 2018; Appendix S1). Iron status was assessed at recruitment. For women who became pregnant, a venepuncture sample was collected for measurement of iron status and malaria infection at a study antenatal visit scheduled at around 13–16 weeks gestation (ANC1) and at a second scheduled study visit between 33 and 36 weeks gestation (ANC2). At delivery the placenta was analysed for malaria parasites. From ANC1 onwards women received standard antenatal care including daily iron and folic acid supplements until delivery (60‐mg iron, 400‐μg folic acid daily). All women received a first dose of intermittent preventive treatment with sulphadoxine pyrimethamine (IPTp) at ANC1 if gestational age was >13 weeks. Women ≤ 13 weeks gestation, if positive for malaria by rapid diagnostic test (RDT) (Bioline SD, Malaria Antigen *Pf* detecting *P. falciparum* histidine‐rich protein 2), were treated with oral quinine. A second scheduled IPTp‐SP dose was provided through routine antenatal care. Gestational age was estimated by ultrasound examination at ANC1 with a FF Sonic UF‐4100 (Fukuda Denshi) scanner, with measurement of crown rump length in the first trimester and by biparietal diameter, femur length and abdominal circumference afterward.

### 2.3 Newborn assessments

At delivery study nurses examined babies within 24–48 h of delivery, and recorded birthweight, using an electronic scale to within 10 g (SECA 384, Hamburg, Germany, precision ± 5 g for weights < 5000 g, ±10 g above 5000 g). Delayed umbilical cord clamping was routinely practised. Following hospital delivery, placental biopsies (2.5 × 1 cm) were excised from fetal and maternal sites at mid‐distance between umbilical cord insertion and the placental border and placed in 10% neutral buffered formalin. Rural health centres were also trained and equipped to take placenta samples. Placental biopsies were not available from home deliveries.

### 2.4 Child assessments

In the wet season in October 2013, mothers who had delivered and remained in the study area were invited to have their offspring assessed at the Clinical Research Unit of Nanoro (CRUN) Health Clinic. The follow‐up child assessment was performed by experienced paediatric nursing research staff and included: medical and vaccination history (BCG and polio at birth; pentavalent diphtheria, tetanus, pertussis, hepatitis B, *Haemophilus influenzae* type B and polio at 2, 3 and 4 months; measles at 9 months; yellow fever at 12 months). Health cards were examined and reports of recent illness, fever and drug intake within the last 2 weeks were checked. Anthropometric measurements were performed in duplicate by two independent observers and mean values computed. Children were weighed to the nearest 10 g with an electronic scale (SECA 384, Hamburg, Germany, precision ± 5 g for weights < 5000 g, ±10 g above 5000 g). Length was measured to the nearest 5 mm using a measuring mat (SECA 210, Germany) and mid‐upper arm circumference to the nearest mm using a circumference tape (SECA 201, Germany). Clinical examination was completed with temperature. A venepuncture blood sample was collected. The volume of blood sampled differed by weight, 1 ml in EDTA and 4 ml in a dry tube for infants ≥ 5600 g and 0.5 and 3.0 ml, respectively, for lower weights. This was used for malaria microscopy and RDT for malaria, Hb, serum iron biomarkers (ferritin, transferrin receptor and zinc protoporphyrin), C‐reactive protein (CRP) and a reserve filter paper blood spot. Children diagnosed with malaria, or any other concurrent health problem, received free treatment according to National Guidelines, with appropriate follow‐up.

### 2.5 Laboratory procedures

Blood samples were transported within 3 h from the clinic to the central project laboratory at CRUN. Sera aliquots were stored at −80°C. Hb was measured (Sysmex automated analyser) and ZPP by fluorometry (Aviv Biomedical) on fresh whole blood. Anaemia in children ≥ 6 months was defined as Hb < 11 g dl^−1^. Plasma ferritin and TfR were measured using mean values from duplicate ELISA samples (Spectro Ferritin S‐22 and TFC 94 TfR, RAMCO Inc.) and CRP by ELISA (EU59131IBL, GmbH). Intra‐assay coefficients of variation (CVs) were all <10%. Ranges for normal controls were ferritin, 69.1–114.7 μg L^−1^; sTfR, 4.2–5.9 mg L^−1^; CRP, 5–8 mg L^−1^. Definitions of iron status were based on (1) adjusted ferritin using the internal regression correction approach (Namaste et al., 2017), allowing for inflammation as described by Mei et al. (2017), or (2) the ratio of sTfR (mg L^−1^) to log_10_ ferritin (μg L^−1^), which assesses both stored and functional iron and is possibly less affected by inflammation. Values > 5.6 in children derive from the cut‐offs sTfR > 8.3 μg ml^−1^ and ferritin < 30 μg L^−1^. This best predicted iron deficient bone marrow stores using the same assay as in the present study, in an area of high malaria transmission (Phiri et al., 2009). Body iron stores (BIS) (mg kg^−1^) using the regression‐adjusted ferritin estimate were calculated using the equation derived by Cook, Flowers, and Skikne (2003): body iron (mg kg^−1^) = −[log_10_ (1000 × sTfR/ferritin) − 2.8229]/0.1207 [39]. Low BIS was defined as <0 mg kg^−1^. Plasma hepcidin was measured by competitive ELISA at an International Reference Laboratory (Kroot et al., 2010). A malaria RDT was performed. Malaria parasite density was obtained from the mean count of two independent readers counting the number of asexual parasites per 200 white blood cells in a thick blood film stained with 3% Giemsa, assuming a white cell count of 8000/μl. For discrepant findings (positive/negative; more than twofold difference for parasite densities ≥ 400/μl; >log_10_ if <400/μl), a third independent reading was made, with the mean of the two closest observations accepted as the true value.

### 2.6 Placental histology

After the delivery of the placenta, it was placed in a receptacle fetal side upwards. Using scissors, a biopsy of 2.5 cm × 1 cm was excised at mid‐distance between the insertion of the umbilical cord and the placenta border and placed into a prefilled specimen container with 10% neutral buffered formalin (CellStor Pot, CellPath Ltd. Newtown SY16 4LE, UK). A 1 cm cross‐section from the umbilical cord was cut with scissors at about 5 cm from the insertion and placed into the same receptacle. After turning the placenta in order to expose the maternal side upwards, a second biopsy of about the same size was excised at half distance between the centre and the border of the placenta and placed into a second container with formalin. A 10 cm × 10 cm piece of the membrane was cut with scissors and placed into the same container.

Formalin fixed specimens were stored for up to 3 months at room temperature in an air‐conditioned room (20°C) at the CRUN laboratory. After transport to the department of pathology at the National University Hospital Yalgado Ouedraogo in Ouagadougou, tissue samples were processed by experienced technicians according to standard histopathological procedures.

Samples were embedded in paraffin wax following standard methods. Membranes were rolled to cylinders in order to obtain sufficient material to be cut and embedded. For each set of samples, paraffin sections 3 to 5 μm thick were placed on two slides, the fetal side of the placenta together with the cord on one slide, the maternal side of the placenta together with the membrane on another slide. Slides were prepared in duplicate to allow for different staining. De‐paraffinised sections were stained with haematoxylin and eosin and Giemsa stain. Histological examination of all samples was done by a specialised senior pathologist with light microscopy and under polarised light. Histological classification of placental malaria was based on the different significance of haemozoin and parasitised RBC in the intervillous space of the maternal side of the placenta as described by Ismail et al. (2000): acute infection (only parasites and minimal haemozoin deposition in the macrophages but not fibrin), chronic infection (parasites and haemozoin deposition), past infection (haemozoin usually mixed with fibrin but no parasites) and no infection. Chorioamnionitis was graded histologically following the Redline‐classification (Redline, 2002).

### 2.7 Statistical analysis

The sample size was determined from formal power calculations for the malaria trial endpoints (Gies et al., 2018). The number of children available for assessment per study arm is shown in Figure S1 (see Appendix S2). The primary analyses presented here are comparisons of prespecified child malaria‐related outcomes and placental malaria in babies by trial arm on an intention to treat basis. Iron and inflammation biomarkers and placental chorioamnionitis were prespecified exploratory outcomes. Preterm was defined as a live birth or stillbirth that took place at least 20 but before 37 completed weeks. Fetal growth restriction (SGA) was defined as birthweight below the 10th centile for gestation and gender, indicated by standard reference data (Villar et al., 2014). Clinical malaria was defined as fever or history of fever (≥37.5°C) in the previous 48 h with parasitaemia. Outcomes were summarised by median (interquartile range) or *N* (%) and compared between treatment arms using ordinary or logistic regression models adjusting for child sex and age at assessment. Age was fitted with a cubic spline function with 5 degrees of freedom, with sensitivity analyses confirming that this was a sufficient representation of the non‐linear age relationships. Results are presented as odds ratios for categorical outcomes or differences between arms for continuous variables with 95% CI. Where appropriate, outcomes were log transformed and the presented effect size can be interpreted as a ratio between arms.

Malaria prevalence by age in various subgroups was visualised using the fitted probabilities from a logistic regression model using a cubic spline representation with the degrees of freedom selected to capture the main features of the age trends without spurious artefacts. Shading around the fitted lines to indicate ±1SE, and rugplots showing the location of positive and negative values (tick marks along top and bottom axes) were added where these did not obscure the presentation.

The associations between infant/maternal factors and infant malaria were assessed using logistic regression models for malaria outcomes against the relevant factors, adjusting for age and sex as above. The associations between iron biomarkers and malaria were visualised as scatterplots of the relationship between the biomarker and age for the children with and without malaria, with cubic spline regression lines added for each group. All analyses were performed in the R statistical environment version 3.6.

### 2.8 Ethical considerations

This child outcome analysis was conducted as a safety assessment of malaria risk in the first born offspring of young mothers enrolled in a randomised periconceptional trial of weekly iron and folic acid supplementation (PALUFER). The study received ethical approvals in Burkina Faso; National Ethics Committee (CERS Ref 015‐2020/CE‐CM) and the Comité d'Ethique pour la Recherche en Santé du Centre Muraz (015‐2010/CE‐CM); the United Kingdom Research Ethics Committee, Liverpool School of Tropical Medicine (LSTM/REC protocol 10‐55); the Institutional Review Board of the Institute of Tropical Medicine (IRB/AB/AC/016) and the Antwerp University Hospital Ethics Committee, Belgium (EC/UZA). The trial was registered with Clinicaltrials.gov on 27 September 2010: https://clinicaltrials.gov/show/NCT01210040.

## 3 RESULTS

### 3.1 Participants

During the trial 478 pregnancies occurred, with 348 known deliveries occurring before the survey period (Figure S1 in Appendix S2). Following exclusions due to out‐migration, stillbirths and neonatal and infant deaths, there were 262 babies eligible for this study. Of these, 180 (69% of those eligible) were contacted, assessed and included in the primary analyses presented here.

### 3.2 Maternal and child characteristics by trial arm

Maternal characteristics at delivery and offspring characteristics by trial arm are shown in Table 1. Maternal characteristics of children surveyed did not differ from those of children lost to follow‐up (see Table S1 in Appendix S2). Maternal iron biomarkers did not differ by trial arm and prevalence of maternal iron deficiency based on low BIS at ANC2 was 16%. Prevalence of placental malaria (active and past infection) was 86% in supplemented women and 81% in controls. Babies born to iron supplemented women had shorter median gestational age and lower mean birthweight (Brabin, Gies, et al., 2019). The median age of children surveyed was 9.1 months (range 1–22 months), 20% were undernourished (*Z* score ≤ 2SD), 2.2% were referred for severe malnutrition (*Z* score ≤ 3SD) and three children had received recent haematinics. Malaria and iron biomarker outcomes are outlined in Table 2. In children ≥ 6 months, 69% were iron deficient (based on a sTfR/log ferritin ratio > 5.6), 33% had low BIS and 98% were anaemic. In children ≥ 6 months, low BIS prevalence was higher than in younger children < 6 months (33% vs. 6%, *P* < 0.001). Mean Hb concentration, iron biomarkers levels or anaemia prevalence in children did not differ between trial arms, nor were there significant differences for any malaria‐related outcomes. Median CRP concentration increased from 2.7 mg L^−1^ in children ≤ 6 months to 6.3 mg L^−1^ for older children (*P* < 0.001), but values did not differ by trial arm.

**TABLE 1:** Child and maternal characteristics by trial arm

Characteristic	Iron	Control
Child		
Median age, months (IQR)	9.6 (4.6–14.4)	8.4 (3.2–13.6)
Age 6 months, *n*/*N* (%)	29/96 (30)	34/84 (40)
Male, *n*/*N* (%)	48/96 (50)	37/84 (44)
Median weight, kg (IQR)	6.9 (5.7–8.4)	7.3 (5.7–8.2)
Undernutrition (*Z* score < 2), *n*/*N* (%)	23/96 (24)	13/84 (15)
Median birthweight, g (IQR)	2756 (2459–3022)	2896 (2518–3065) (4 missing)
Median gestational age, days (IQR)	269 (260–275) (8 missing)	273 (265–280) (4 missing)
Preterm < 37 weeks, *n*/*N* (%)	21/88 (24)	8/80 (10)
SGA, *n*/*N* (%)	23/88 (26)	23/80 (29)
Maternal parameters at ANC1		
Median BMI, kg m^−1^ (IQR)	21.3 (19.9–22.6) (9 missing)	20.8 (20.2–21.6) (3 missing)
Median Hb, g dl^−1^, *N* (IQR)	10.1 (9.3–11.0) (8 missing)	10.2 (9.1–11.1) (2 missing)
Median sTfR/log ferritin ratio^a^ (IQR)	3.2 (2.5–4.2) (8 missing)	3.0 (2.4–4.4) (4 missing)
Median body iron stores, mg kg^−1^ (IQR)	6.5 (4.5–9.1) (9 missing)	6.9 (4.0–9.7) (4 missing)
Median hepcidin, nmol L^−1^ (IQR)	2.2 (0.8–5.3) (9 missing)	2.8 (0.7–7.0) (2 missing)
Median CRP, mg L^−1^ (IQR)	5.6 (2.01–12.2) (9 missing)	4.1 (0.9–12.2) (2 missing)
Malaria parasite positive, *n*/*N* (%)	46/87 (53)	45/82 (55)
Maternal parameters at ANC 2		
Median sTfR/log ferritin ratio^a^ (IQR)	4.1 (3.0–6.3) (20 missing)	4.6 (3.0–6.8) (11 missing)
Median body iron stores, mg kg^−1^ (IQR)	4.7 (1.0–7.9) (21 missing)	4.0 (0.8–6.5) (13 missing)
Median hepcidin, nmol L^−1^ (IQR)	0.9 (0.4–3.0) (21 missing)	0.7 (0.3–2.5) (11 missing)
Median CRP, mg L^−1^ (IQR)	4.08 (1.19–12.11) (21 missing)	2.11 (1.14–4.88) (12 missing)
IPTp received (≥2), *n*/*N* (%)	69/96 (72)	66/82 (80)
Maternal parameters at delivery		
Placental malaria: None, *n*/*N* (%)	8/58 (14)	11/59 (19)
Placental malaria: Active, *n*/*N* (%)	18/58 (31)	11/59 (19)
Placental malaria: Past, *n*/*N* (%)	32/58 (55)	37/59 (63)
Chorioamnionitis, *n*/*N* (%)	25/58 (43)	30/56 (54)

Abbreviations: BMI, body mass index; CRP, C‐reactive protein; Hb, haemoglobin; IQR, interquartile range; IPTp, intermittent preventive antimalarial treatment with sulphadoxine pyrimethamine in pregnancy; SGA, small for gestational age; sTfR, serum transferrin receptor. Control group includes one pair of twins with birthweights < 2500 g.

^a^ Log (sTfR/log ferritin ratio) uses value for adjusted ferritin.

**TABLE 2:** Child outcomes by trial arm

Outcome	Iron	Control	Difference^a^ (95% CI)	*P* value
Malaria related				
RDT positive, *n*/*N* (%)	69/95 (73) (1 missing)	60/84 (71)	0.8 (0.3–1.9)	0.60
Microscopy positive, *n*/*N* (%)	36/95 (38) (1 missing)	26/84 (31)	1.4 (0.7–2.7)	0.31
Clinical malaria, *n*/*N* (%)	14/96 (15)	9/84 (11)	1.3 (0.5–3.3)	0.62
Parasite density per mm^3^ (*N*) (IQR)	6201 (36) (823–15780)	3900 (851–17496) (26)	0.72 (0.25–2.06)	0.53
Fever (≥37.5°C), *n*/*N* (%)	13/96 (14)	7/84 (8)	1.5 (0.5–4.2)	0.44
Fever in last 14 days, *n*/*N* (%)	26/96 (27)	24/84 (29)	0.8 (0.4–1.5)	0.44
Malaria treatment last 14 days, *n*/*N* (%)	17/96 (18)	17/84 (20)	0.8 (0.3–1.7)	0.48
Number of malaria episodes since birth, *N* (IQR)	1 (0–2) (2 missing)	1 (0–2)	−0.1 (−0.4 to 0.2)	0.51
Any illness in last 14 days, *n*/*N* (%)	49/96 (51)	43/84 (51)	0.8 (0.4–1.6)	0.60
Anaemia and iron biomarkers				
Median Hb, g dl^−1^ (*N*) (IQR)	8.7 (95) (7.4–9.7) (1 missing)	9.2 (7.6–10.0) (1 missing)	−0.1 (−0.6 to 0.3)	0.53
Hb < 11 g dl^−1^ in children ≥ 6 months age, *n*/*N* (%)	64/66 (97) (1 missing)	49/49 (100) (1 missing)	NA	NA
Median CRP, mg L^−1^ (*N*) (IQR)	6.2 (2.1–19.0) (2 missing)	4.6 (1.1–13.4) (1 missing)	1.4 (0.8–2.3)	0.23
Median adjusted ferritin, μg L^−1^ (IQR)^b^	64.8 (20.6–199.3) (11 missing)	57.4 (19.7–211.8) (9 missing)	1.2 (0.8–1.8)	0.31
Median sTfR, μg ml^−1^ (IQR)	17.5 (9.8–24.6) (3 missing)	14.4 (9.5–20.3) (2 missing)	1.1 (0.9–1.3)	0.41
sTfR/log ferritin ratio (IQR)^c^	9.0 (4.5–14.6) (12 missing)	8.0 (5.2–11.2) (10 missing)	1.0 (0.8–1.2)	0.78
Median ZPP, μmol mol^−1^ haem (IQR)	196 (146–319) (1 missing)	206 (143–257) (1 missing)	1.0 (0.9–1.2)	0.47
Median body iron stores, mg kg^−1^ (IQR)	3.7 (0.1–8.5) (12 missing)	3.8 (0.2–8.1) (10 missing)	0.6 (−0.8 to 2.1)	0.40

*Note*: NA, insufficient data to compute OR.

Abbreviations: CRP, C‐reactive protein; Hb, haemoglobin; IQR, interquartile range; sTfR, serum transferrin receptor; RDT, rapid diagnostic test; ZPP, whole blood zinc erythrocyte protoporphyrin.

^a^ Difference for continuous variables, odds ratio for dichotomous variables. Where variables are log transformed, the difference in logged values can be interpreted as a ratio.

^b^ Ferritin adjusted with internal regression correction using common slope.

^c^ Log (sTfR/log ferritin ratio) uses adjusted ferritin.

### 3.3 Child malaria prevalence and iron biomarkers for combined trial arms

**FIGURE 1:**
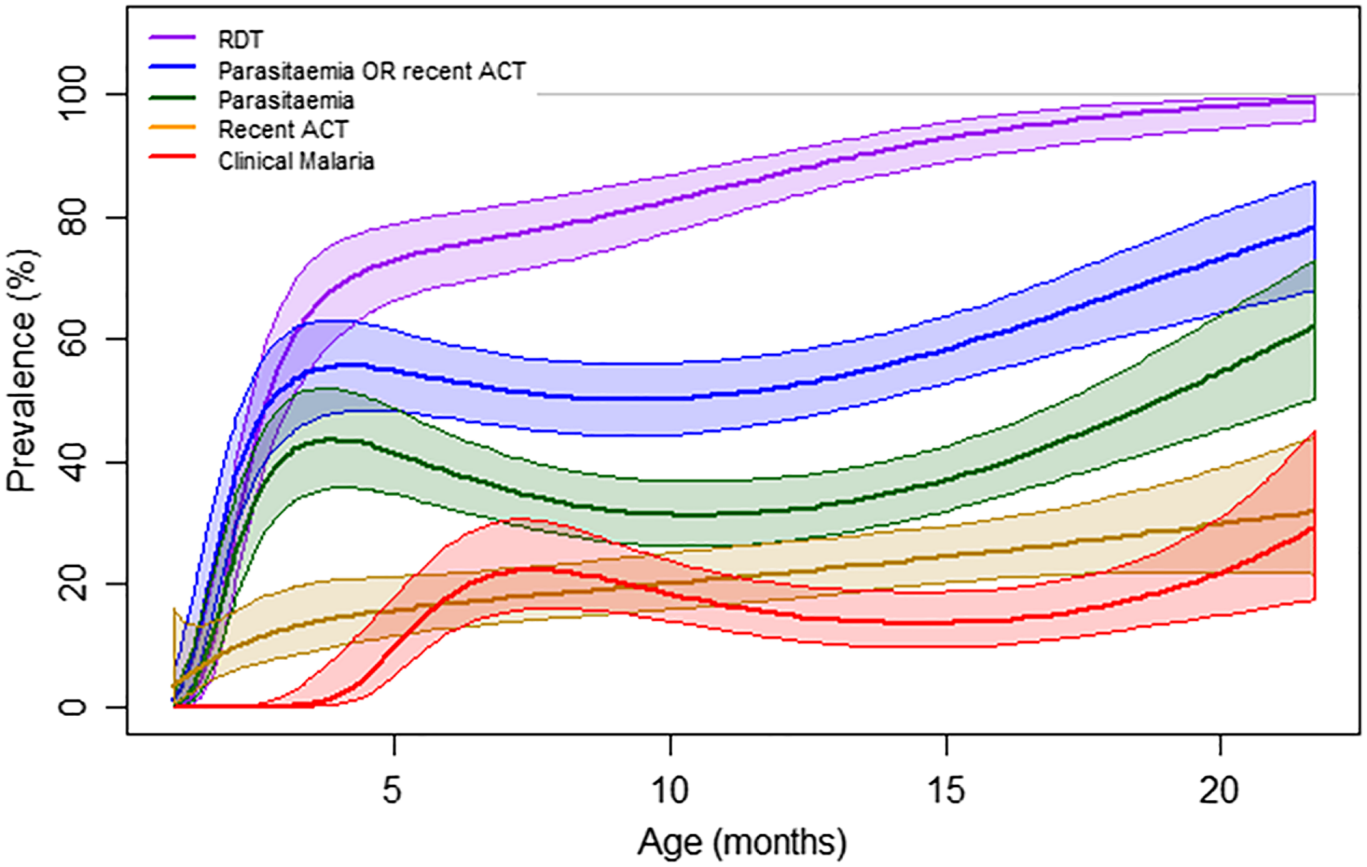
Malaria prevalence by age in children under 2 years. Shaded areas are ±1SE around the fitted line. RDT, rapid malaria diagnostic test; ACT, recent antimalarial combination treatment. Clinical malaria was defined as fever or history of fever (≥37.5°C) in previous 48 h with parasitaemia

Parasite prevalence increased from low values at 1 month to approximately 45% by 4 months, with positive RDT prevalence increasing to 70% at this age. Clinical malaria prevalence increased to 20% by 7 months. Prevalence of all malaria infection parameters progressively increased with age (Figure 1).

**FIGURE 2:**
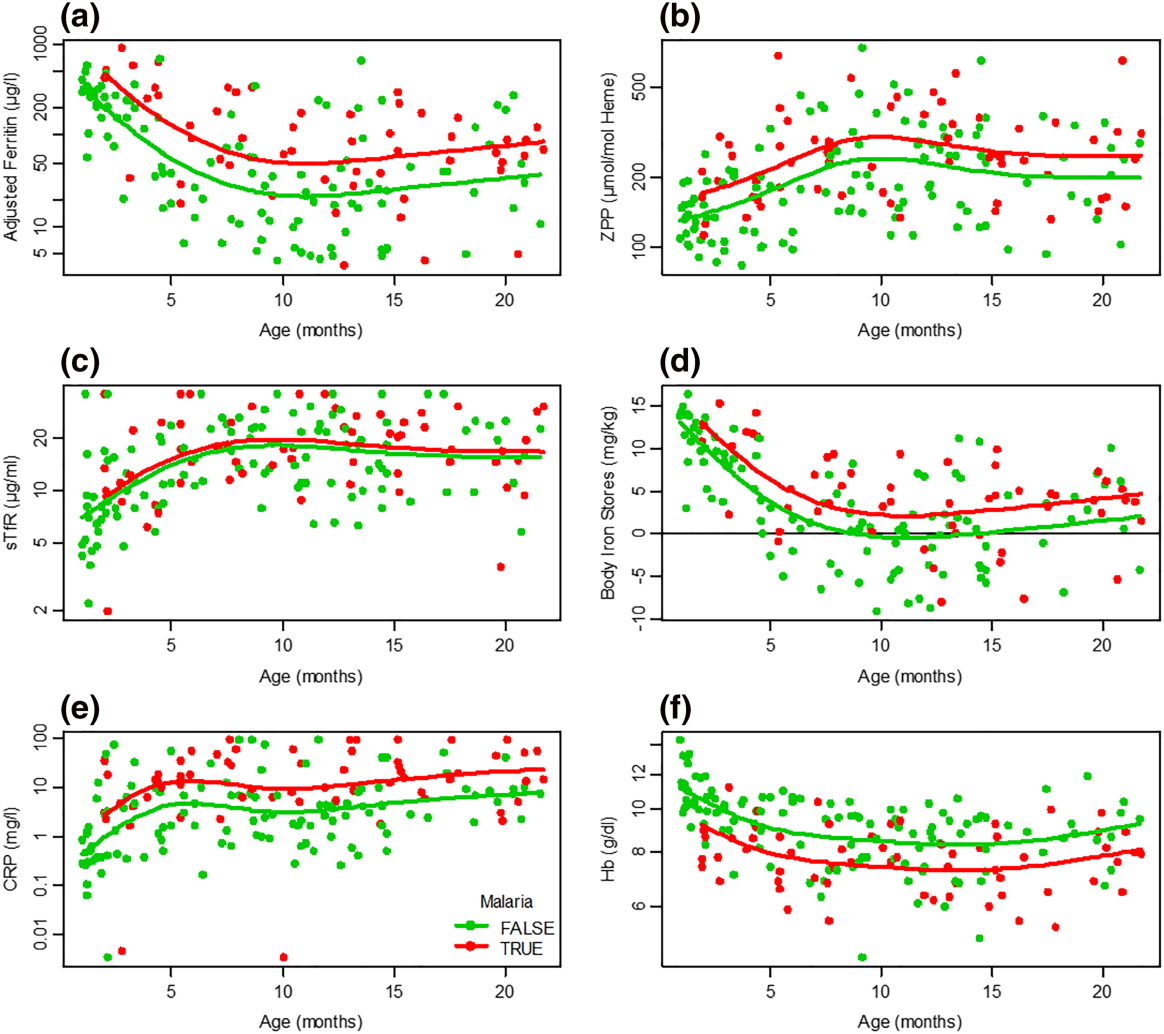
Age‐specific child iron biomarkers and malaria parasitaemia. (a) Adjusted ferritin; (b) ZPP, whole blood zinc protoporphyrin; (c) sTfR, serum transferrin receptor; (d) BIS, body iron stores (horizontals line is zero body iron stores); (e) CRP, C‐reactive protein and (f) Hb, haemoglobin. Red line: malaria parasitaemia present; green line: malaria parasitaemia absent

The age‐specific pattern of child iron biomarkers is shown in Figure 2 in relation to malaria parasitaemia. Malaria infection was associated at all ages with lower Hb, higher CRP concentration and higher values for adjusted ferritin (*P* < 0.001) and BIS (*P* < 0.001). Similar iron biomarker differences were seen in relation to parasitaemia and/or recent malaria treatment (see Figure S2). Parasitaemia risk was higher with evidence of childhood iron repletion (Table 3). The risk estimate (OR) for malaria parasitaemia with BIS (per mg kg^−1^ increase) was 1.13 (95% CI 1.04–1.23, *P* = 0.002) and for parasitaemia and/or recent malaria treatment 1.18 (95% CI 1.09–1.29, *P* < 0.001). Higher zinc protoporphyrin concentration was associated with malaria infection (*P* = 0.003). Gestational age at delivery, SGA, and child undernutrition were not associated with childhood malaria risk.

**TABLE 3:** Predictors of child malaria in combined trial arms

Predictor	*N*	Current parasitaemia		Current parasitaemia or recent malaria treatment	
		Odds ratio (95% CI)	*P* value	Difference^a^ (95% CI)	*P* value
Birth and nutrition					
Sex: Male	85/179	0.7 (0.3–1.3)	0.22	1.1 (0.6–2)	0.86
Birthweight (per kg)	175	0.5 (0.2–1.2)	0.13	0.6 (0.2–1.4)	0.20
Gestation (per week)	168	1.0 (0.8–1.3)	0.81	1.1 (0.9–1.3)	0.41
Preterm < 37 weeks	29/168	1.2 (0.5–3.2)	0.67	0.9 (0.4–2.3)	0.87
Child undernutrition (*Z* score < 2)	36/179	1.3 (0.6–2.8)	0.56	1.5 (0.7–3.5)	0.29
Child anaemia and iron biomarkers					
Hb	178	0.5 (0.4–0.7)	<0.001	0.5 (0.4–0.7)	<0.001
Adjusted ferritin^b^	160	4.0 (1.9–8.4)	<0.001	5.6 (2.6–12.2)	<0.001
Log (sTfR)	175	2.2 (0.5–10.5)	0.30	2 (0.4–9.2)	0.36
Log (sTfR/log ferritin ratio)^c^	158	0.4 (0.1–1.6)	0.19	0.2 (0–0.7)	0.015
Log (ZPP)	178	14.1 (2.3–85.7)	0.003	20.5 (3.3–129.2)	<0.001
Body iron stores	158	1.13 (1.04–1.23)	0.002	1.18 (1.09–1.29)	<0.001
Log (CRP)	177	2.9 (1.7–5.1)	<0.001	3 (1.7–5.1)	<0.001
Maternal parameters at ANC1					
BMI	168	1.2 (0.9–1.4)	0.15	1 (0.8–1.3)	0.75
Hb	170	1.0 (0.8–1.3)	0.98	1.1 (0.8–1.4)	0.50
Log (sTfR/log ferritin ratio)^c^	167	1.0 (0.2–5.1)	0.97	1.3 (0.2–6.4)	0.78
Body iron stores	167	1.01 (0.93–1.11)	0.79	0.99 (0.91–1.08)	0.81
Log (hepcidin)	169	0.9 (0.5–1.6)	0.65	1.1 (0.6–2.1)	0.65
Log (CRP)	169	1.6 (0.9–2.8)	0.096	1.2 (0.7–2.1)	0.41
Malaria parasite positive	91/169	2.0 (1.0–4.3)	0.063	1.4 (0.7–2.8)	0.36
Maternal parameters at ANC2					
Log (sTfR/log ferritin ratio)^c^	146	1.1 (0.2–6.6)	0.94	0.7 (0.1–4)	0.69
Body iron stores	146	1.02 (0.93–1.12)	0.70	1.04 (0.95–1.14)	0.39
Log (hepcidin)	148	1.2 (0.6–2.4)	0.64	1.2 (0.6–2.4)	0.56
Log (CRP)	147	2.1 (1.1–3.9)	0.020	1.7 (0.9–3)	0.070
IPTp received (≥2)	135/177	1.4 (0.7–3.2)	0.35	1.1 (0.5–2.3)	0.80
Placental pathology					
Placental malaria: Noninfected	19	Reference		Reference	
Placental malaria: Active infection^d^	29	5.8 (1.0–32.5)	0.042	10 (1.8–56.2)	0.008
Placental malaria: Past infection^e^	69	3.2 (0.7–14.1)	0.13	10.3 (2.2–48)	0.003
Chorioamnionitis	55/114	0.4 (0.1–1.0)	0.038	0.7 (0.3–1.7)	0.44

Abbreviations: ANC1/ANC2, scheduled first and second study antenatal visits; BMI, body mass index; CRP, C‐reactive protein; Hb, haemoglobin; IPTp: intermittent preventive antimalarial treatment in pregnancy with sulphadoxine pyrimethamine; SGA, small for gestational age; sTfR, serum transferrin receptor; ZPP, whole blood zinc protoporphyrin.

^a^ Difference for continuous variables, odds ratio for dichotomous variables. Where variables are log transformed, the difference in log values can be interpreted as a ratio.

^b^ Ferritin adjusted with internal regression correction using common slope.

^c^ Log (sTfR/log ferritin ratio) uses value for adjusted ferritin.

^d^ Active infection: includes acute infection (only parasites and minimal haemozoin deposition in the macrophages but not fibrin) and chronic infection includes parasites and haemozoin deposition.

^e^ Past infection: haemozoin usually mixed with fibrin but no parasites.

**FIGURE 3:**
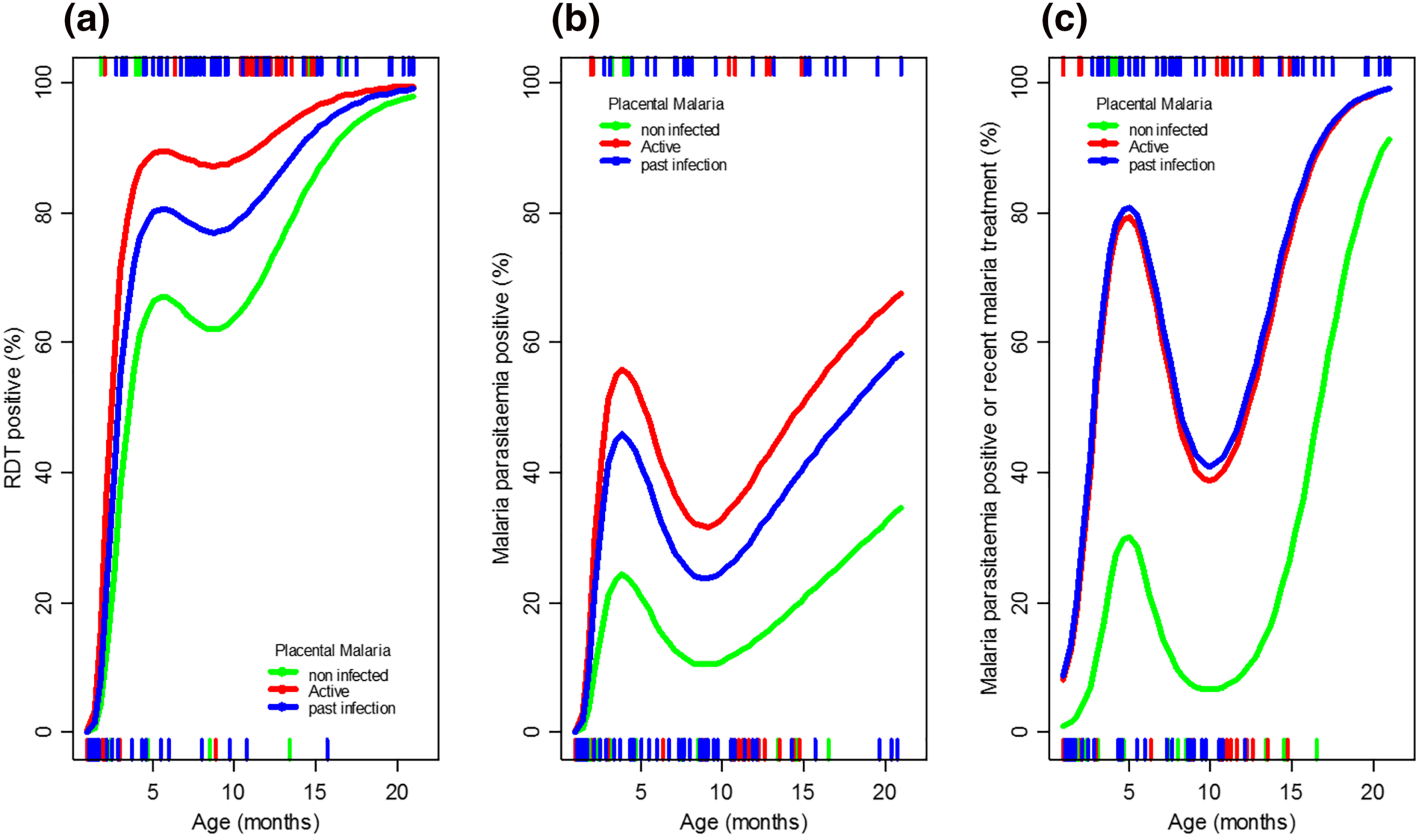
Child malaria infection prevalence by placental malaria category. Red line: active placental malaria present; blue line: past placental malaria present; green line: no placental malaria present. (a) Rapid diagnostic test positive, (b) malaria parasitaemia positive and (c) malaria parasitaemia positive and/or recent treatment for malaria. Active indicates acute infection (only parasites and minimal haemozoin deposition in the macrophages but not fibrin) and chronic infection (parasites and haemozoin deposition). Past indicates previous infection (haemozoin usually mixed with fibrin but no parasites). Rugs along the axes indicate the positive (upper axis) and negative (lower axis) case

Child malaria infection prevalence grouped by categories of active and past placental infection are shown in Figure 3. Placental malaria infection was associated with higher malaria infection prevalence at all child ages. The difference in childhood malaria infection risk associated with placental malaria occurred after about 3–4 months of age and persisted through the second year. The difference in childhood malaria infection prevalence associated with active or past placental infection was ≤10%, with higher estimates for active placental infection. Effect estimates for this risk associated with placental malaria are shown in Table 3 which summarises child and maternal parameters associated with current parasitaemia in children. An odds ratio for child parasitaemia of 5.8 (95% CI, 1.0–32.5, *P* = 0.042) was associated with active placental infection but was higher for parasitaemia and/or recent malaria treatment (OR 10.0, 95% CI, 1.8–56.2, *P* = 0.008). Past placental malaria infection also predicted child parasitaemia and/or recent malaria treatment (OR 10.3, 95% CI 2.2–48, *P* = 0.003). Malaria parasitaemia at ANC1 marginally increased the risk of childhood parasitaemia (OR 2.0, 95% CI 1.0–4.3, *P* = 0.063). In children, parasitaemia risk was higher with evidence of maternal inflammation based on raised CRP (OR 2.1, 95% CI 1.1–3.9, *P* = 0.020) in the third trimester. Chorioamnionitis at delivery was associated with reduced risk of child parasitaemia (*P* = 0.038), although for child parasitaemia or recent malaria treatment this reduction was not significant.

## 4 DISCUSSION

Periconceptional iron supplementation made no difference to placental malaria parasite prevalence, or in children, to malaria‐related outcomes or iron biomarker values. When combining trial arms, increased malaria infection prevalence was seen in children with better iron status. There was increased childhood malaria infection risk following active and past placental malaria, indicating that earlier gestational malaria was also a risk factor for malaria in young children, although the active infection had cleared by the time of delivery.

The absence of a difference in child malaria outcomes or iron status by trial arm is consistent with the findings that periconceptional iron supplementation did not improve maternal iron status. There was good adherence to weekly supplementation (79% iron; 80% control; Gies et al., 2018), and the lack of effect of maternal iron supplementation was attributed to poor maternal iron absorption due to the high prevalence (>40%) of chronic untreated asymptomatic malaria parasitaemias (Brabin, Tinto, & Roberts, 2019). In principle screening for iron deficiency, prevalence would be implemented before introduction of routine supplementation programmes. WHO in 2020 suggested that a prevalence of 5–19.9% iron deficiency, based on ferritin concentrations, might be considered a mild public health problem (World Health Organisation, 2020). Based on adjusted ferritin alone (<15 μg L^−1^), prevalence at ANC1 in our study was 12.8% (Diallo et al., 2020). Using BIS as an indicator, 16% of mothers in the last trimester and subsequently 6% of their infants aged ≤6 months had values < 0 mg kg^−1^. Their relative iron repletion could predispose to earlier onset malaria infection in infants. Routine iron supplementation to young menstruating nulliparous women should not be recommended without prior effective malaria control as the majority could be iron replete (Brabin et al., 2020). In programmatic terms providing periconceptional iron routinely to these women, most of whom were not iron deficient, also increased risk of preterm birth and on this basis should not be recommended (Table 1; Brabin, Gies, et al., 2019).

Younger babies would be more likely to have recrudescences from congenital malaria which, in this area, affected 10% of newborns (Natama et al., 2017). Malaria parasite prevalence (with or without prior treatment) and RDT positivity in children were, with the exception of ZPP, strongly associated with biomarkers of better iron status and iron repletion at all ages (Table 3). ZPP concentration was higher in children with malaria, but its specificity in children, as well as in pregnant women, is reduced secondary to the anaemia of inflammation (Asobayire, Adou, Davidson, Cook, & Hurrell, 2001; Senga, Koshy, & Brabin, 2012; Stoltzfus et al., 2000). Preterm delivery and low birthweight lead to higher iron requirements for growth and are factors likely to contribute to the high prevalence of iron deficiency and anaemia in children older than 6 months in this area (Brabin, Gies, et al., 2019; Domellöf, 2017). Malaria contributes an added risk for anaemia and infection had been experienced by approximately 80% of children reaching 6 months of age. Comparable high iron deficiency and anaemia prevalence has been reported in the Eastern region of Burkina Faso in children 6–12 months of age (Bliznashka, Arsenault, Becquey, Ruel, & Olney, 2020).

An early peak in malaria prevalence occurred at around 4 months of age followed by uniformly rising prevalence with increasing age. An early peak in prevalence was similarly associated with placental infection in a cohort of Cameroonian children (Le Hesran et al., 1997). However in a cross‐sectional survey an early peak may be an artefact since age reflects intensity and cumulative exposure to malaria. Children at 4 months may have had more exposure (almost a full season at the time of the survey), compared with younger children who have had less exposure, and older ones who have developed some acquired immunity from a previous season's exposure (Natama, Rovira‐Vallbona, Somé, et al., 2018). This would occur in children of mothers with and without placental malaria, so does not explain the higher malaria prevalence in those with placental malaria. A recent meta‐analysis of 11 studies found an overall malaria risk in young children (adjusted hazard ratio 1.46, 95% CI 1.07–2.0, *P* < 0.001) associated with malaria in pregnancy but via indeterminate mechanisms (Park et al., 2020). Placental malaria may influence primarily risk for the first infant malaria episode (Bouaziz et al., 2018; Le Hesran et al., 1997; Le Port et al., 2011), and better child iron status could increase subsequent malaria risk (Georgiadou et al., 2019) and possibly risk of nonmalarial infections (Natama, Rovira‐Vallbona, Sorgho, et al., 2018; Rachas et al., 2012).

Malaria risk in young children is also influenced by other maternal factors, although this study found no association with gestational age, use of IPTp, SGA or child undernutrition. Evidence for an association between IPT in pregnancy and malaria in infants is limited (Kakuru, Staedke, Dorsey, Rogerson, & Chandramohan, 2019). Higher malaria risk has been reported in a single study of SGA infants, but gestational age was not assessed by ultrasound and the difference was marginal (Agbota, Polman, et al., 2019). Maternal–fetal immunological interactions are important (Feeney, 2020; Jagannathan, 2018). Innate immune modulation by placental malaria is described in infants from this study area in Burkina Faso (Natama, Moncunill, et al., 2018). Immunological interactions are especially relevant in primigravidae who are at increased risk of malaria in high transmission areas. In the present study, all women were primigravidae and more than 90% were adolescent (Brabin et al., 2020). The lower risk of child malaria observed in this study with chorioamnionitis may be attributable to treatment of related maternal symptoms of vaginal discharge from genital infection with metronidazole, which has antimalarial effects and hence could reduce placental parasite load (Pallangyo, Minjas, & Sarda, 1986). Metronidazole for lower genital infections with vaginal discharge was available free of charge both before and during pregnancy and 8.2% reported a discharge at least once during pregnancy (Brabin et al., 2017; background data, Appendix S1).

Lack of adjustment for malaria transmission intensity is an important limitation of the present study, as for nearly all published studies, which may be confounded by shared maternal and child exposures to infected mosquitoes (Kakuru, Staedke, Dorsey, Rogerson, & Chandramohan, 2019). Adolescent malaria has been associated with high gametocyte prevalence in Burkina Faso, which would influence the reproductive rate and infection transmission (Ouédraogo et al., 2010). Malaria infection risk could relate to an effect of ferritin supply on gametocyte fecundity and parasite ontogeny in the mosquito (Geiser, Conley, Elliott, Mayo, & Winzerling, 2015). If mosquitos have higher malaria gametocyte rates (enhanced by better iron substrates in their blood meal), then the reproduction rate of malaria transmission increases. This would impact on the local mosquito population and increase malaria infant exposures. For this reason, infants of a largely iron replete adolescent population could be at increased malaria infection risk. Younger women may also provide different child care practices than older mothers which may impact their child's malaria exposure and treatment. Iron requirements for adolescent growth and for pregnancy and neonatal iron are competing factors (Young et al., 2010), which underlines the need for longitudinal studies of infection risk characterising iron biomarkers from preconception.

No previous studies have longitudinally assessed both maternal and child iron status as determining factors for childhood infection risk in areas of high malaria transmission. Lack of efficacy of the primary intervention in mothers precluded better understanding of the effects of supplementation on the iron status of their offspring. Strengths of the present study include assessment of gestational age by ultrasound, lack of confounding due to maternal parity or age, inclusion of key determinants of maternal and child malaria and completion of this cross‐sectional survey during the peak month of malaria transmission (Natama et al., 2018). The findings should be generalizable to comparable areas with high malaria transmission. A limitation was the reduced sample size for the child survey. This was predicated by the primary safety trial endpoints in mothers and the requirement to complete the survey during the peak malaria transmission month. The sample size for the child safety survey was reduced due to perinatal losses (Gies et al., 2018), as well as migration outside the study area which contributed to failure to attend for delivery, or led to postnatal migration and failure to attend for the child survey. Mobility was high among girls as they married and moved to join their husbands in nonstudy areas (Campaoré, Gies, Brabin, Tinto, & Brabin, 2018). Village deliveries and those occurring en route to the hospital reduced placental biopsy samples. Attrition and child nutritional and age profiles were equivalent between trial arms.

## 5 CONCLUSIONS

High prevalence of placental and child malaria was associated with better iron status in young mothers and children. Intensified efforts are needed to reduce risk of placental malaria which should, in turn, reduce risk of malaria and anaemia in young children.

## CONFLICTS OF INTEREST

The authors declare that they have no conflicts of interest.

## CONTRIBUTIONS

BB and SG designed the research. BB was the Principal Investigator for the main randomised controlled trial and recipient of the grant. SG, SD, BB and HT conducted the field research. SD and OL conducted and supervised the laboratory research. SAR analysed the data. BB and SR wrote the paper. All authors reviewed and approved the final manuscript.

## Supporting information


**Appendix S1.** Further Trial Details

**Figure A1.** Participant flow diagram
**Table A1.** Antenatal characteristics at ANC1 of mothers of children lost to follow‐up and of children included in follow‐up survey
**Figure A2.** Age specific child iron biomarkers in relation to malaria parasitaemia or malaria treatment

## Data Availability

Until placed in a public repository, study data can be requested from the corresponding author and made available following an end user data agreement and sponsor approval.
